# Automated verbal autopsy: from research to routine use in civil registration and vital statistics systems

**DOI:** 10.1186/s12916-020-01520-1

**Published:** 2020-03-09

**Authors:** Riley H. Hazard, Mahesh P. K. Buddhika, John D. Hart, Hafizur R. Chowdhury, Sonja Firth, Rohina Joshi, Ferchito Avelino, Agnes Segarra, Deborah Carmina Sarmiento, Abdul Kalam Azad, Shah Ali Akbar Ashrafi, Khin Sandar Bo, Violoa Kwa, Alan D. Lopez

**Affiliations:** 1 Melbourne School of Population and Global Health, The University of Melbourne, Carlton, Victoria Australia; 2 The George Institute for Global Health, UNSW Sydney, Newtown, New South Wales 2042 Australia; 3 Department of Health, Manila, Philippines; 4 Directorate General of Health Services, Dhaka, Bangladesh

**Keywords:** Verbal autopsy, Civil registration and vital statistics, Cause of death, Bangladesh, Myanmar, Papua New Guinea, Philippines

## Abstract

**Background:**

The majority of low- and middle-income countries (LMICs) do not have adequate civil registration and vital statistics (CRVS) systems to properly support health policy formulation. Verbal autopsy (VA), long used in research, can provide useful information on the cause of death (COD) in populations where physicians are not available to complete medical certificates of COD. Here, we report on the application of the SmartVA tool for the collection and analysis of data in several countries as part of routine CRVS activities.

**Methods:**

Data from VA interviews conducted in 4 of 12 countries supported by the Bloomberg Philanthropies Data for Health (D4H) Initiative, and at different stages of health statistical development, were analysed and assessed for plausibility: Myanmar, Papua New Guinea (PNG), Bangladesh and the Philippines. Analyses by age- and cause-specific mortality fractions were compared to the Global Burden of Disease (GBD) study data by country. VA interviews were analysed using SmartVA-Analyze-automated software that was designed for use in CRVS systems. The method in the Philippines differed from the other sites in that the VA output was used as a decision support tool for health officers.

**Results:**

Country strategies for VA implementation are described in detail. Comparisons between VA data and country GBD estimates by age and cause revealed generally similar patterns and distributions. The main discrepancy was higher infectious disease mortality and lower non-communicable disease mortality at the PNG VA sites, compared to the GBD country models, which critical appraisal suggests may highlight real differences rather than implausible VA results.

**Conclusion:**

Automated VA is the only feasible method for generating COD data for many populations. The results of implementation in four countries, reported here under the D4H Initiative, confirm that these methods are acceptable for wide-scale implementation and can produce reliable COD information on community deaths for which little was previously known.

## Background

Informed health policy to reduce premature mortality and improve population health requires reliable estimates of the leading causes of death for the entire population, not just those who die in hospitals where physicians are available to certify the cause of death (COD). However, comparatively few countries benefit from reliable and timely evidence about who dies of what, particularly lower- and middle-income countries (LMICs) where the epidemiological transition is likely to be advancing rapidly and where the need for such information is arguably the most acute [1]. Recent assessments of the quality of data from national civil registration and vital statistics (CRVS) systems worldwide suggest that only about one quarter of all countries (55–60) have functioning systems that can adequately support policy formulation and evaluation [2, 3]. In LMICs, most deaths typically occur at home and are often not registered, or if they are, are not certified by a trained medical practitioner. Rather, the COD, if notified at all, is generally assigned by non-medical untrained personnel, resulting in a large fraction of ill-defined or otherwise vague diagnoses that are of little value for guiding policy to control the leading causes of death in the population [4].

Traditionally, the development of a functional CRVS system has taken countries such as Sweden, the UK and Australia several decades, if not centuries, to achieve and has generally required sufficient levels of national wealth, education and supply of physicians before reliable statistics on causes of death could be produced [5]. Circumventing this long delay by developing and implementing low-cost, alternative and efficient methods to generate the essential health intelligence for planning has thus become an urgent and fundamental challenge for health measurement strategies. Given the lack of trained physicians in many LMICs, verbal autopsy (VA) is the only practical alternative for collecting information on the leading causes of death in such populations, and how they are changing.

VA involves trained local workers (often non-physician health care workers) administering structured, symptom-based questionnaires to the caretakers of the deceased and transferring the responses to a database where they are analysed using a method for assigning the COD. Historically, physicians assigned a COD based on the responses to the questionnaire [6]. Recent advances in methodological research and computer technology have automated this process whereby computer algorithms analyse the responses to the questionnaires and assign a COD, with significant cost and time savings [7]. Automated diagnostic methods have the advantage of standardisation, circumventing comparability problems arising from variations in physician judgement across populations. Moreover, automated diagnostic methods do not burden physicians with additional administrative tasks that detract from their primary role of delivering health care. A variety of algorithms have been proposed, some making extensive use of data collected in specialised demographic and health surveillance sites, primarily in Africa, where the processes for interview and data analysis are well established [8–11]. These sites have demonstrated that community-based COD systems using VA are certainly feasible, but the potential for VA to be used on a routine basis in national CRVS systems to provide essential information on broad population COD patterns has only recently been evaluated under the Bloomberg Philanthropies Data for Health (D4H) Initiative [12–15].

In this paper, we report on experiences using SmartVA, a specific data collection and diagnostic tool that has been demonstrated to perform as well as, if not better than most alternative methods, including physician-assigned COD [16]. We draw a number of important conclusions from our experience in adapting the automated VA method from a research environment to a software tool and demonstrate the considerable potential for automated VA to be routinely applied in national CRVS systems to dramatically improve the evidence base on who dies of what in LMICs.

## Methods

### Country data collection

The D4H Initiative aims to strengthen CRVS systems in countries. During the process of D4H country work plan development, the need for better information on community deaths, or a sub-set of deaths not currently captured through medical certification of COD, was identified as the highest priority, with most countries involved in D4H committed to the implementation of VA to obtain this information. A 5-day training curriculum with associated materials was developed and subsequently adapted to country needs [17–19]. Since the aim is to use this information to strengthen CRVS systems, the general module of the VA questionnaire, concerned with administrative information (such as date and place of death, usual residence, etcetera), was also adapted by countries to allow such data to be incorporated into the current CRVS system.

**Fig. 1:**
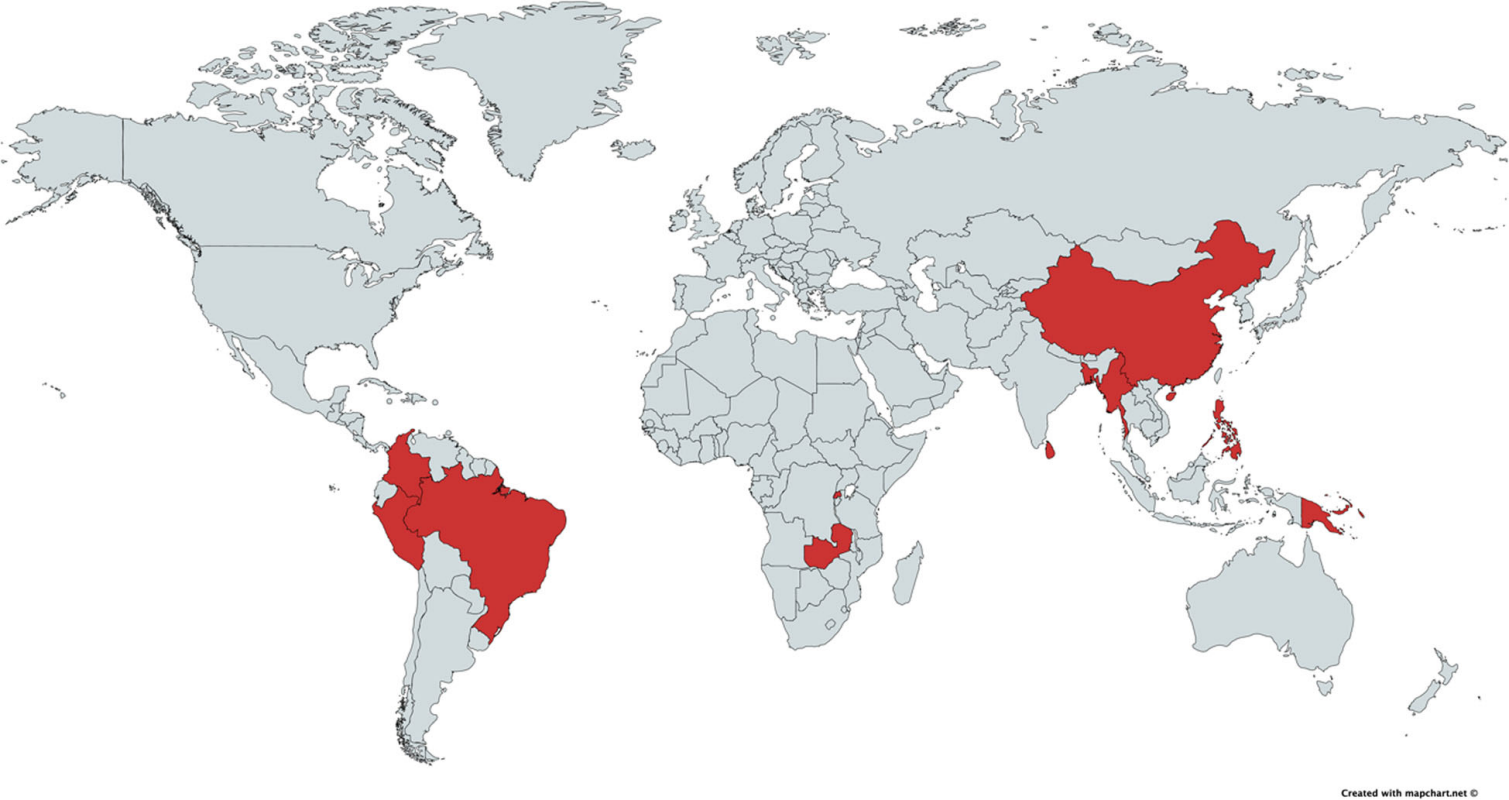
Map of countries where SmartVA has been applied

SmartVA has been translated and applied in several D4H intervention countries, including Bangladesh, Brazil, China, Colombia, Papua New Guinea (PNG), the Philippines, Myanmar, Peru, Rwanda, Solomon Islands, Sri Lanka and Zambia (Fig. 1). VA implementation ideally follows a number of stages, through a pre-test, pilot, demonstration stage and gradual scale-up [12]. Consequently, different numbers of VAs were collected and processed using this methodology, reflecting the different stages of VA programme implementation across the D4H countries. To illustrate the challenges and achievements of this methodology to rapidly improve knowledge on causes of death in rural populations at low cost, we report on the implementation of SmartVA in four countries: Myanmar, PNG, Bangladesh and the Philippines. These countries were selected to highlight the application of SmartVA in countries at varied stages of health statistical development.

### Verbal autopsy questionnaire

For VA interview application in D4H countries, we used the Open Data Kit (ODK) software, the most widely used electronic data collection tool for VA. Data collection using ODK Collect on Android tablets and data storage with ODK Aggregate have greatly improved data quality [20]. The use of a common platform like ODK, with the ability for translation of the VA questionnaire, also enabled the use of automated VA in non-English-speaking countries.

An important aspect of VA questionnaire design is the time taken to conduct a VA interview. For routine application in a country, which might well entail tens of thousands of VAs every year, it is desirable that the interview be as short as possible so as to minimise interviewer and respondent fatigue and distraction, while still reliably capturing essential diagnostic information. The shortened version of the Population Health Metrics Research Consortium (PHMRC) questionnaire was used in SmartVA. This questionnaire was systematically shortened by about 50% from a longer version that was used in research sites using item reduction methods, without a significant decline in diagnostic performance [21]. Empirical evidence from the application of the shortened SmartVA questionnaire suggests that the interview can be completed in 20–22 min, on average, with a further 3–5 min required for the open narrative section [7, 22].

### Tariff VA diagnostic algorithm

The Tariff VA diagnostic algorithm was originally developed by the PHMRC based on the premise that certain symptoms are more strongly associated with certain specific causes than others [23]. The resulting ‘tariff’ scores for each symptom-cause pair should, in principle, provide sufficient information to adequately discriminate among various potential causes of death, depending on the pattern of responses to the VA symptom questionnaire, and other information provided at the time of interview. In other words, the diagnostic procedure should be entirely data-driven (by the strength of the observed tariff scores) and not dependent on expert opinion. Subsequent developments of the Tariff algorithm (Tariff 2.0) improved the diagnostic accuracy of the method, by assessing predictive performance against a large ‘Gold Standard’ diagnostic database of over 12,000 cases where the COD was reliably known, by refinements to methods used to incorporate the ‘open narrative’ (free-flowing text in the respondent’s words about events leading up to death), and incorporating a recalibration of thresholds used to determine some specific diagnoses based on experience with field application of the method [9, 24]. Full details on the development and performance characteristics of the Tariff method can be found elsewhere [9, 16, 21, 23].

Comparative performance studies, where alternative diagnostic methods were validated against the PHMRC Gold Standard database, demonstrated that the Tariff method was able to correctly predict COD fractions at the population level, arguably the most relevant information for policy, about 77% of the time, compared with 63–69% for other automated diagnostic methods such as InterVA, and 68% when physicians were used to diagnose VA questionnaires [25].

### Verbal autopsy software

We used SmartVA-Analyze version 2.0 to implement the Tariff 2.0 method with the SmartVA questionnaire [26]. This version of SmartVA-Analyze has some important differences from previous versions, such as more user-friendly outputs for analysing and interpreting results. The primary use of SmartVA-Analyze is to generate cause-specific mortality fractions (CSMFs) to identify the leading causes of death (as a fraction) in the community.

There are two main outputs from SmartVA-Analyze: individual cause predictions and population distribution of causes of death (i.e. CSMFs). Individual cause predictions provide a COD for each VA interview completed, along with those for which a cause assignment could not be made with sufficient certainty based on the symptom pattern reported by the family (an ‘undetermined’ COD). The population distribution of causes of death, or the CSMFs, aggregates the individual predictions from the VA interviews and, in addition, redistributes the undetermined causes of death among the causes that can be diagnosed based on the evidence from the GBD and the cause distribution of undetermined cases from a comparison with gold standard diagnoses [9]. This redistribution is done in two ways. Firstly, a VA with an undetermined COD is fractionally distributed among all VA causes, with weights proportional to the likelihood that the particular cause was diagnosed as undetermined in the gold standard database. Certain deaths (such as pneumonia) are more likely to be reported as an undetermined COD because the condition is inherently more difficult to diagnose using VA methods than an event such as a road traffic accident. Secondly, this fractional redistribution weight is averaged with a proportional redistribution weight selected according to the GBD age-sex COD distribution for the country based on the alignment with covariates and other determinants of the epidemiological environment of a population that the GBD measures. This redistribution is done at the population level since the primary purpose of VA is to correctly understand the COD patterns in populations, not individuals.

A more recent use of VA, as an aid for physicians, prompted the development of the second software application: ‘SmartVA Auto-Analyze’. In the Philippines, it is mandatory for municipal health officers to write a death certificate for all non-facility deaths—even those for which they had little or no contact with the deceased. The software offers a standardised, logical, symptom-based platform to elicit useful diagnostic information from the family. The output of Auto-Analyze differs from that of SmartVA-Analyze because it produces only individual results. Physicians conduct the VA interview, and Tariff 2.0 presents them with the top three most likely causes of death along with the basic demographic characteristics of the deceased and a full list of all symptoms endorsed by the family. The physician reviews this information and, using any other information as available from the family, completes the death certificate—choosing either one of the Tariff-assigned causes of death or an alternative cause. Auto-Analyze has been configured in English, Spanish and Chinese and is currently being trialled in selected countries, including China, to standardise and enhance procedures for diagnosing home deaths.

### Assessing the plausibility of CSMFs from VA

Routine collection of VA as part of the CRVS system is a new but important challenge for most countries, and as such, the data collected need to be understood and interpreted carefully. To assist countries with this task, we have proposed a series of steps that countries can and should follow to assess the plausibility of their VA CSMFs [27]. This method involves firstly describing the VA implementation area and assessing the extent to which it is similar or dissimilar to a (usually national) comparator. For instance, if geographic features, population age distribution or the epidemiological profile of the VA population differs from the national average, CSMFs will be expected to differ. Other factors to consider include completeness of information on death reporting in the VA population (are all deaths registered, and do they all have a VA) and whether the age-sex distribution of death makes sense given the profile of the VA implementation area. Some assessment of the VA CSMFs against a comparator dataset is useful once the characteristics of both datasets are understood. Under D4H, a tool to accompany these guidelines has been developed to assist and guide countries in how to systematically review their VA data for plausibility [28].

### Global Burden of Disease

In many LMICs, there are few sources of data that can be used to compare against population COD information produced through a routine application of VA. In order to assess whether the CSMFs from the application of SmartVA to rural populations produced plausible results, we compared the CSMFs and age distributions of deaths from SmartVA to the findings from the Global Burden of Disease Study 2017, which give estimated COD patterns for each country, by sex and age [3]. The GBD study is a systematic, scientific and comprehensive collaboration to estimate patterns, levels and trends in the causes of death and disability in countries for over 350 diseases and injuries for each year since 1990. The estimates are modelled based on the existing mortality, morbidity and covariate data, corrected for known biases, and thus represent the predicted levels and patterns of mortality given the covariate values for factors likely to affect specific diseases and injury outcomes, such as education, income, smoking prevalence and diet. While the GBD estimates are not strictly comparable to the outputs from SmartVA, since the latter are generally limited to community deaths only, the comparisons are still likely to be meaningful given that community deaths are likely to account for the vast majority of deaths in these countries. In all our VA country samples, the numbers of neonatal and child deaths are too low to conduct such a comparison. Therefore, we present results for adult deaths only, which provide sufficient numbers for comparison.

## Results

In each of the D4H countries, the process of implementation involved a formative phase, including stakeholder consultation, formation of a country-specific technical working group, process mapping to understand the CRVS process^1^ and translation and cognitive testing of the questionnaire to ensure that respondents understood the intent of the questions [12]. Each country carried out a small-scale pilot followed by a larger-scale implementation of SmartVA in a defined sample. Monitoring and evaluation of each phase were ensured by the technical working group. The pre-test, pilot and demonstration phases were evaluated using mixed-methods including both qualitative (focus group discussions with interviewers) and quantitative (analysis of causes of death) methods.

### Myanmar

In Myanmar, the majority of deaths (84%) occur in the community, with COD assigned through lay reporting to a midwife at the time of registering the death. This information is entered into the CRVS database and integrated with medically certified deaths. Not only does this results in vague or erroneous COD information for the majority of deaths that happen in the community that *are* registered, but often deaths are not registered at all, leaving the government with very poor mortality data on which to base their health policy. The Ministry of Health and Sports (MoHS) and the Central Statistical Organization (CSO) of Myanmar partnered with the D4H initiative to increase registration and improve information on the cause of community deaths by using automated VA, utilising basic health staff (midwives and public health supervisors), mandated to collect this information. The SmartVA questionnaire was translated into the Myanmar language and loaded into the ODK Collect application on tablets. A pre-test (300 VA interviews) in 3 townships^2^ in 2 regions and 1 state was conducted in 2016, and a pilot in 14 townships (11,238 VA interviews) in the same 3 states and regions was completed in 2017 [29]. Minor adjustments to the questionnaire and process for scale-up (training, monitoring and feedback) were informed by lessons from these early stages of implementation.

Improved death registration practices and automated VA were scaled up in 2018 to include 42 townships across the country, with at least 2 townships in each state and region and Nay Pyi Taw Union Territory. These 42 townships covered a population of over 8.1 million, or around 16% of the total population of the country. They were selected to be broadly representative of the population of Myanmar. Within these 42 townships, VA was conducted on all notified community deaths that occurred between January and December 2018. Basic health staff and their supervisors were trained in SmartVA methods for 5 days by national and township VA master trainers from the MoHS and the CSO. Data from the field was sent to a central server at the CSO, downloaded, cleaned and analysed monthly using SmartVA-Analyze, with output from the analysis sent to the respective townships. Formal evaluations were jointly conducted with local stakeholders, in July 2018 and January 2019, to assess the plausibility of the findings in the context of GBD estimates for Myanmar and local knowledge about the epidemiological environment [30].

### Papua New Guinea

Mortality surveillance in PNG is at an early stage of development, almost entirely derived at present from health facility discharge summaries, the Discharge Health Information System (DHIS). The discharge diagnosis is recorded in the same manner for deaths as for discharges back to the community and does not allow for multiple causes as in part A of the international standard medical certificate of COD. The proportion of all deaths in PNG captured by the DHIS is approximately 10–15%, as the majority of deaths occur in the community and are rarely registered. There is no national mortality surveillance system capturing data for community deaths.

PNG is currently rolling out an electronic National Health Information System (eNHIS) that aims to collect individual-level data from all health centres and hospitals in the country using tablet devices with automated upload to a server at the National Department of Health. The initial design of eNHIS included a drop-down menu for discharges, including deaths, that was not adequate for reporting underlying COD. The collaboration with D4H aimed to pilot systems for a comprehensive mortality surveillance system in PNG using eNHIS. This involved testing strategies for community-level reporting of deaths to enable notification and VA by health staff, as well as collaboration with the developers of eNHIS to enable reporting of medical certification of COD data and VA data through the electronic system.

VA data collection in PNG was implemented in three districts in 2018. The districts were selected to reflect the diverse geographical and political landscapes across PNG but were not intended to be a representative sample. Rather, they were selected to identify different challenges and potential solutions, to guide subsequent, wider country implementation. The three districts were Alotau, Milne Bay Province; Talasea, West New Britain Province; and Tambul-Nebilyer, Western Highlands Province. Training was provided for notifying agents in each district to notify deaths to their nearest health centre, and VAs were conducted by trained health workers: health extension officers, nurses and community health workers. An evaluation of preliminary findings was conducted in the first quarter of 2019 by the Department of Health and National Burden of Disease Technical Advisory Group, which suggested a substantially different COD pattern for the country than that estimated by the GBD Study.

### Bangladesh

Bangladesh has no functioning routine CRVS system for registering deaths and determining the COD. With support from the D4H Initiative, Bangladesh thus established collaborative mechanisms between the health sector at the community level and the local civil registrar and provided training to community health workers (staff with non-medical background) to report deaths and conduct VA during their routine household visits.

A VA pilot intervention was first introduced in Kaliganj Upazila (local administrative area with a population of 304,600) in 2016. The shortened version of the SmartVA questionnaire was translated and tested in local Bengali language, and an electronic local language version was installed on Android tablets for administering VA. Community-based health workers (CHW) were trained and mandated to identify deaths in the community and help families complete the registration forms and collect the associated certificates during their routine visits, as well as notify the events to the local registration office for official registration within 45 days of occurrence. When death is identified, and following the mourning period, the CHW arranges an appointment to meet with the family to conduct a VA interview using the tablet.

This pilot, known as the ‘Kaliganj Model’, was successful, with a very high coverage of death registration (> 90%) and VA administration [31]. Following this success, the Government of Bangladesh scaled up the model to apply in additional sub-districts (Upazila) in the country. From 2017 to March 2019, VA was rolled out in 13 sub-districts, including all 5 sub-districts of the Gazipur District and at least 1 sub-district selected purposefully from each of the 8 divisions of the country, covering a total population of 4.8 million. VAs were administered in 13 sub-districts, and VA interview data were electronically transferred from the community to an ODK aggregate server.

### Philippines

In the Philippines, SmartVA was used as a decision support tool to assist municipal health officers (MHOs) in completing death certificates for deaths that occur in the community. A technical working group identified 13 sites where a pre-test was conducted in 1 language to assess feasibility and acceptability. The intervention included training 127 MHOs in SmartVA Auto-Analyze and medical certification of COD. Next, the intervention was scaled up to 50 municipalities across 6 regions of the country using 3 different languages. A mixed-methods evaluation was performed using COD data and group discussions with the MHOs and the Department of Health. MHOs and information technology (IT) staff were trained by D4H trainers over 3 days. Of the 5644 deaths, SmartVA was used to certify 4419 (78%); for the remainder, MHOs considered that there was sufficient information available in the medical records to certify the cause without SmartVA. SmartVA was found to be readily acceptable by the MHOs and family members and has been incorporated into the routine workflow of the MHOs.

### Cause of death data from applications of SmartVA

The characteristics of the VA adult deaths collected in each of the four countries are shown in Table 1. Myanmar had the largest sample of deaths, followed by Bangladesh, the Philippines and PNG. In all four countries, the number of male deaths outnumbered female deaths. In Myanmar and the Philippines especially, females died at older ages than males.

**Table 1:** Characteristics of VA interviews processed by SmartVA-Analyze for Myanmar, Bangladesh, PNG and the Philippines (adult deaths)

Country (year of data)	Number of adult cases	Male to female ratio in the sample (%)	Age, median (IQR) years
Myanmar (2018)	39,331	56:44	Males, 62 (47–75) Females, 72 (59–82)
PNG (2017)	612	60:40	Males, 54 (38–66) Females, 52 (31–71)
Bangladesh (2017–2018)	12,320	62:38	Males, 66 (53–77) Females, 69 (52–79)
Philippines (2018)	4267	55:45	Males, 65 (54–76) Females, 72 (60–83)

The age distribution of the country VA samples seen in Table 2 was evaluated by comparing with the GBD age estimates. In Myanmar and Bangladesh, the proportion of deaths generally increased with age, closely mirroring the pattern of GBD deaths. The age distribution of deaths in PNG was generally younger, with the highest proportion of deaths occurring between ages 50 and 69, similar to the age distribution of deaths suggested by the GBD comparator data, reflecting the comparatively high mortality among younger adults in the country than elsewhere.

**Table 2:** Age distribution of VA deaths compared to the GBD age distribution of deaths, selected countries

Age group (years)	Myanmar		PNG		Bangladesh		Philippines	
	VA, *n* (%)	GBD (%)	VA, *n* (%)	GBD (%)	VA, *n* (%)	GBD (%)	VA, *n* (%)	GBD (%)
12–19	511 (1.3)	2	29 (4.7)	3.7	160 (1.3)	2.3	60 (1.4)	2.1
20–29	1219 (3.1)	4.2	82 (13.4)	9.6	382 (3.1)	3.2	115 (2.7)	4.7
30–39	2793 (7.1)	5.6	68 (11.1)	13.1	678 (5.5)	4	196 (4.6)	6.3
40–49	4208 (10.7)	9.1	80 (13.1)	17.5	1220 (9.9)	6.8	341 (8)	9.7
50–59	5664 (14.4)	15.1	88 (14.4)	19.8	1885 (15.3)	13.5	585 (13.7)	15.3
60–69	7591 (19.3)	20.5	111 (18.1)	19	2526 (20.5)	20.8	870 (20.4)	21.2
70–79	7906 (20.1)	21	82 (13.4)	12.8	2710 (22)	23.4	990 (23.2)	20.8
80+ years	9400 (23.9)	22.5	53 (8.7)	4.5	2587 (21)	26	1109 (26)	19.9
Unknown	39 (0.1)	0	19 (3.1)	0	172 (1.4)	0	0 (0)	0
Total	39,331 (100)	100	612 (100)	100	12,320 (100)	100	4267 (100)	100

**Fig. 2:**
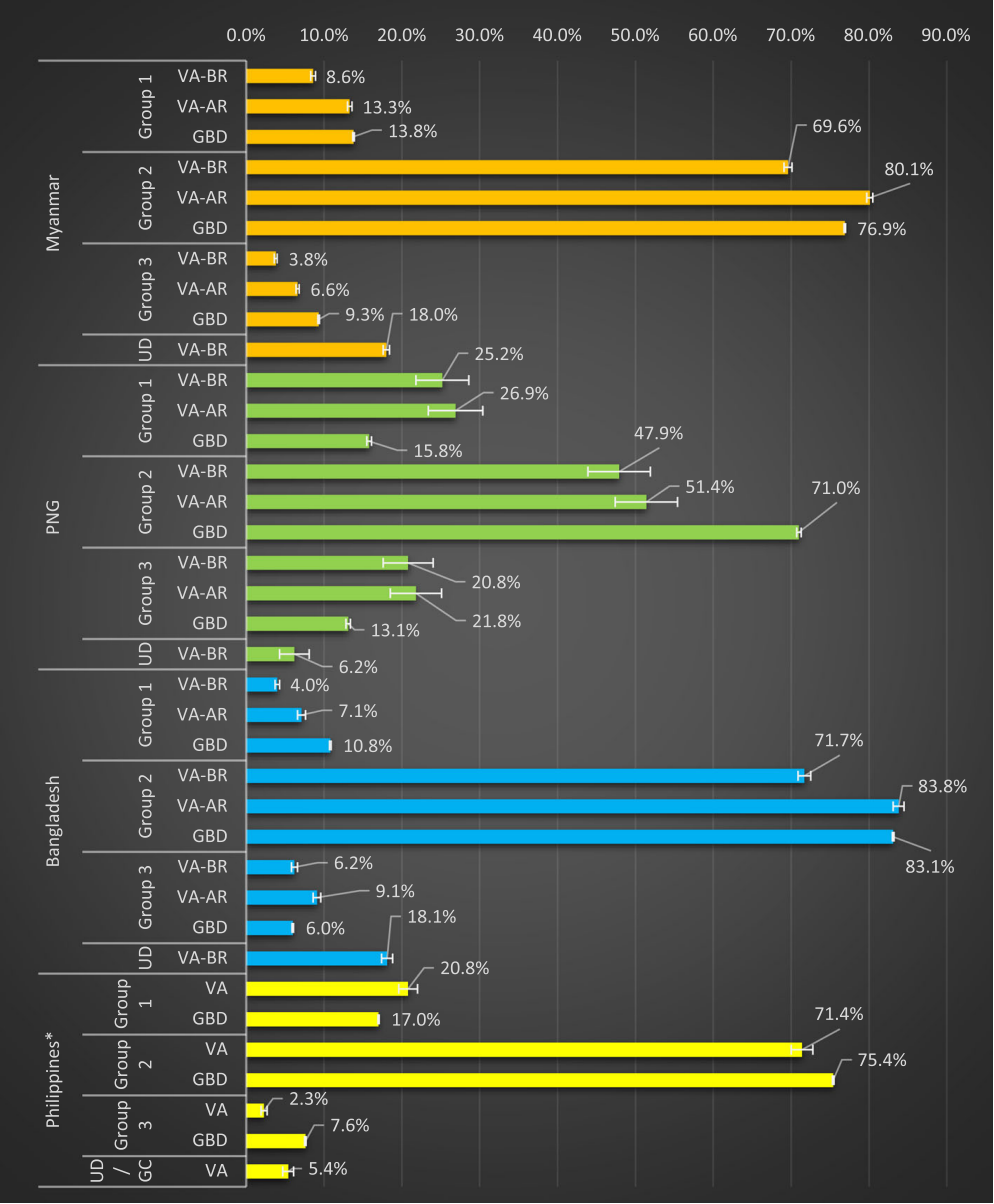
Distribution of level-1 GBD cause categories of VA deaths compared to GBD estimates. Group 1: communicable, maternal, and nutritional; group 2: non-communicable diseases; group 3, injuries. *Distribution of level-1 GBD cause categories after mapping the VA-assisted causes of death compared to GBD estimates for the Philippines, excluding 239 deaths which had been categorised as alcohol-related (3 deaths), other respiratory (4 deaths), undetermined (146 deaths), and garbage codes (for 86 deaths). BR, before redistribution; AR, after redistribution; UD, undetermined; GC, garbage code

For policy, it is important to understand at what stage a country is in its epidemiological transition. A convenient metric to do so is the distribution of deaths according to the three broad cause groups of the GBD: communicable, maternal, neonatal and nutritional conditions; non-communicable diseases (NCDs); and injuries. The cause distribution of SmartVA outputs for the various countries according to these broad cause groups is shown in Fig. 2, with and without undetermined causes reallocated for Myanmar, PNG and Bangladesh. Reallocation was not required in the Philippines as physicians determine the COD for any undetermined cases produced through VA. The 95% confidence intervals were considerably larger for the causes in PNG, where the sample size was only 612, compared to causes in Myanmar and Bangladesh with sample sizes of 39,331 and 12,320, respectively.

Generally, SmartVA predicted similar proportions of deaths in the three broad cause categories compared to the GBD estimates, except for PNG where SmartVA predicted a much lower fraction of NCD deaths among adults than the GBD estimates and considerably more injuries and communicable, maternal and nutritional diseases. This result is in accord with other epidemiological research in PNG and also with a local expert opinion based on the demand for health care services [32]. The VA-assisted CSMF for injuries in the Philippines was less than that predicted by the GBD. This discrepancy may have been due to injury-related deaths undergoing either an autopsy for police investigation or certification in the hospital, rather than inclusion in the VA-assisted sample. For all countries, the ranking of causes was the same for both the undetermined and reallocated causes.

The proportion of undetermined causes of adult deaths (deaths for which there was not sufficient certainty to assign a COD) was around one in six (18%) in Myanmar and Bangladesh (18%), but considerably lower in PNG (6%) and the Philippines (5%). The low fraction of undetermined deaths in PNG and the Philippines most likely reflects the stronger symptom ‘signal’ for communicable and maternal causes in PNG, where these conditions are more common (and so respondents are better at symptom identification), and the direct involvement of physicians in the Philippines in reviewing SmartVA outputs and additional information provided by the family at interview.

## Discussion

Experience with SmartVA under the D4H Initiative suggests that the method can be readily incorporated into routine CRVS systems to reliably identify broad COD patterns in communities, thus serving as an important source of information for health sector priority debates. The tool has also provided more detailed COD information on the probable leading causes of death within each of the broad cause groups, as required for the identification and evaluation of health interventions. SmartVA has proven to be an extremely useful tool in all countries where it has been trialled to generate COD information for populations, where hitherto there was no reliable information about who died of what. While the methodology has been introduced at different stages of implementation in various countries, the four country experiences reported here demonstrate that SmartVA methods are feasible for large-scale collection and analysis of COD data for health policy purposes. The ease of uptake of SmartVA in countries from across the globe highlights its usability and acceptability. A proportion of VAs produce ‘undetermined’ causes of death, but this fraction generally reduces once VA interviewers become more competent in their understanding of VA and in their interview techniques [30], underscoring the critical importance of careful training and evaluation of interviewer performance for any application of VA.

GBD is not a ‘gold standard’, and comparison with other sources of data in a country, as well as the characteristics of the VA population compared to the population that GBD represents, is necessary. Indeed, for PNG, the VA COD results suggest a lower fraction of deaths from NCDs compared with the GBD models, but the discrepancy with GBD is not unexpected by health experts in the country, many of whom believe that GBD has overestimated the progress of the epidemiological transition, which is likely to vary considerably across PNG [32]. For the other countries, age distribution and pattern of broad causes of death predicted by SmartVA were generally consistent with that estimated for the same country by the GBD both with and without undetermined causes reallocated. Notwithstanding the caution of using GBD estimates as a comparison, this does increase the confidence in the utility of the outputs for informing public health policy. In the comparisons with GBD data, it is important to keep in mind that SmartVA has only been applied to home (community) deaths which could be expected to have a different cause composition to deaths that occur in hospitals and other health facilities. In addition, the age distribution of death in PNG was skewed to higher ages in the VA sample compared to GBD. In the absence of a gold standard dataset to validate COD outputs from the two approaches, it is difficult to judge which set of data is likely to be more reliable and, to some extent, that is of lesser importance. What is important is that the Ministry of Health in PNG can now benefit from direct evidence, as opposed to model-driven estimates, on the leading causes of death in a population who do not regularly access health services, and act accordingly.

The SmartVA methodology can be implemented to achieve several health information system objectives. For many countries, routine application of the method in its simplest form, even on a sample of home deaths captured by the CRVS system, will likely yield highly informative and largely unbiased information on the leading causes of death for deaths where there has been little or no contact with the hospital system. Other countries, for example, the Solomon Islands, have used SmartVA to gather information about ‘dead on arrival’ cases at major hospitals where the COD is otherwise unattainable or to improve the diagnostic accuracy of individual death certificates for community deaths. Indeed, SmartVA Auto-Analyze might be the optimal option for automated VA in instances where individual death certificates are still signed by a medical officer, given their diagnostic accuracy is likely to be significantly enhanced, and standardised, by the information derived from the routine application of the SmartVA Auto-Analyze tool. Other hybrid versions, involving non-physicians collecting VA information, with physicians reviewing the results and assigning a COD, have also been successfully trialled. The flexibility of the methods to accommodate local requirements has been an important factor in the success of the intervention in the participating countries.

VA implementation is complex and relies on several phases: translation and transcultural adaptation of the questionnaire and materials; intensive training of the staff allocated to conduct the VA interview; rigorous monitoring and supervision of these staff; and IT capacity at different levels of the system to manage data collection, transfer, analysis and dissemination. The system-level considerations for VA implementation are discussed elsewhere [12, 33]. Government investment and commitment to the process is a pre-requisite for the sustainability of this intervention. The continued commitment of governments currently implementing VA in D4H countries is indicative of the appreciation of the benefits that such cost-effective, reliable and standardised methods can yield for collecting information on community deaths, often for the first time.

In terms of contribution to the strengthening of CRVS systems in the country, VA has provided an impetus for the improvement of community death registration practices that could be replicated in non-VA sites. For example, in the Kaliganj pilot in Bangladesh, the implementation of VA increased death registration from 30% in 2016 to 91% in 2018, and in Myanmar, increases in registration completeness were seen in both pilot (14 townships) and country-wide scale-up (42 townships) [30, 34]. Routine collection and analysis of data from community deaths, the first time such an undertaking has been attempted in some countries, is the first step towards improving vital statistics. Ensuring that such data can be routinely and reliably collected and then integrated with the existing or evolving CRVS infrastructure is another challenge that needs to be met [12].

Several important research and development priorities have emerged from this initial global trial of integrating SmartVA into routine CRVS systems as a means of improving the availability and quality of COD data for policy and planning. The global health utility of SmartVA (and VA software in general) could be greatly improved with the addition of new language versions, and particularly through additional ‘gold standard’ data in order to improve and expand (to other causes) the assessment and validation of VA performance characteristics. VA diagnostic algorithms would also benefit from a more efficient and comprehensive means of incorporating information about local epidemiological environments as well as from improving diagnostic procedures to derive maximum information content from the open narrative section of the interview. Effectively integrating this new source of COD data into current CRVS and health information systems is also a key challenge. This involves consideration of what data management tools are required, such as ‘CRVS/VA dashboards’ in order to monitor data collection; how often, and where, remedial interviewer training should be offered, including the role of online training; and how VA data might be integrated with other sources of mortality data collected by the CRVS system. Efforts to integrate VA into the widely used District Health Information System software (DHIS-2) show promise [35]; however, incorporating this information into existing CRVS systems will require a high level of cooperation between agencies for data sharing and strong IT capacity, something that is often lacking in the statistical systems of developed and developing countries alike.

## Conclusions

Of the 55 million deaths that occur worldwide each year, about 40% go unregistered; another 40% occur in hospitals or health establishments where physicians are available, in principle at least, to certify the COD; and the remaining 20% are notified to authorities but the underlying COD is either unknown or insufficiently specified to be of public health use [3]. Yet, to improve population health for these (mostly) rural communities where most deaths occur at home, efficient mortality reduction strategies require reliable COD information systems, and the only viable means to do so is the routine use of automated VA. It is simply not imaginable that requiring busy rural physicians to do so on a regular basis is sustainable, despite claims to the contrary [36]. Our experience under the D4H Initiative in more than a dozen countries suggests that reliable methods such as SmartVA can readily, cost-effectively and in a timely fashion yield the essential information needed to guide public policy [14, 15]. The results now being produced from the country implementation of SmartVA, illustrated here with data from Myanmar, PNG, Bangladesh and the Philippines, confirm that these methods are acceptable for a wide-scale implementation and can produce reliable COD information on community deaths for which little was previously known. The widespread application and integration of automated VA into national CRVS systems in LMICs would likely lead to a very substantial improvement in the evidence base available for policy and planning and for monitoring progress towards national and global development goals.

## Data Availability

The data that support the findings of this study are available from the civil registration and vital statistics system of each country, but restrictions apply to the availability of these data, which were used under licence for the current study, and so are not publicly available.

## Abbreviations

CHW: Community-based health workers
COD: Cause of death
CRVS: Civil registration and vital statistics
CSMFs: Cause-specific mortality fractions
CSO: Central Statistical Organization
D4H: Data for Health
DHIS: Discharge Health Information System
DHIS-2: District Health Information System
eNHIS: Electronic National Health Information System
GBD: Global Burden of Disease
IT: Information technology
LMICs: Lower- and middle-income countries
MHO: Municipal health officer
MoHS: Ministry of Health and Sport
NCD: Non-communicable disease
ODK: Open Data Kit
PHMRC: Population and Health Metrics Research Consortium
PNG: Papua New Guinea
VA: Verbal autopsy

## Footnotes

1 See paper in this series: *Better data for better outcomes: The importance of business processes management in CRVS systems.*

2 A township in Myanmar refers to a ‘low-level’ local government unit.
